# Items

**Published:** 1881-12

**Authors:** 


### Items.

TUBERCLE: ITS HISTOLOGICAL CHARACTERS, AND ITS RELATION TO THE INFLAMMATORY PROCESS, AS SHOWN IN "TUBERCULOSIS" OF LYMPHATIC GLANDS. By FREDERICK TREVES, F.R.C.S. Read before the International Medical Congress.

The term was here limited to the appearance known as sub-miliary, lymphoid, or primitive tubercle, or tubercular follicle. Tubercle, the author said, represented a certain stage or degree of a peculiar form of inflammation; that developed tubercle was usually chronic, and induced by slight excitation; its exudations were very cellular, and in certain places presented large nuclear-like bodies (Rindfleisch), which might be regarded as almost special to the process; the products resisted absorption, and lingered in the tissues. Non-vascularity of the part was an early feature. Degenerative changes usually ensued, most commonly caseation. If a certain stage of the process were reached, giant-cells appeared, or the so-called tubercle was met with. Above all, this inflammation required for its development the anatomical element of adenoid, or lymphatic tissue. In the tubercle producing process in glands, the first changes are those of simple inflammation. The appearance of giant-cells, or of tubercle proper, indicated a certain stage in the process. The stage might never be reached, and glands, if affected with some intensity, might caseate before any giant-cell or tubercle had been seen. Tubercle occurred in the most chronic or least intense form of the inflammatory process. It represented, in one sense, the highest attainable morbid change. Giant-cells were by no means special to tubercle. They were merely lymph-coagula, and indicated the cessation of all lymph current. In regard to histology, Mr. Treves said that the fibrous material of glands, the seats of tardy inflammation, tended to arrange itself in roundish districts. . If the fibrous matter in these districts were somewhat open, and giant-cells were introduced, the appearance of tubercle might be seen. The reticulum occupying the spot presented no constant appearance, but was modeled precisely upon the arrangement of the fibrous matter in the vicinity. Many of the so-called tubercles had the giant-cell at the periphery, others had no giant-cell, many are oval, or of the most irregular outline. The giant-cell, he urged, was here also merely a lymph-coagulum, that blotted out a certain amount of the recticulum in which it was deposited. As the mass degenerated, the recticulum often came into view, and could be seen to be continuous with the adjacent tissue by means of the giant-cell processes.

---

OFFICIAL LIST OF CHANGE OF STATIONS AND DUTIES OF MEDICAL OFFICERS of the United States Marine Hospital Service, July 1, 1881, to September 30, 1881:

*Bailhache, P. H.*, Surgeon. Detailed as member of Board to examine keepers and crews of Life Saving Stations. September 8, 1881. To inspect the service of stations in Maine, New Hampshire and Massachusetts. September 9, 1881.

*Wyman, Walter*, Surgeon. When relieved of special duty as medical officer revenue-bark "Chase," to rejoin his station, *via* Washington, D. C. August 18, 1881.

*Long, W. H.*, Surgeon. Detailed as Chairman Board of Examiners. September 12, 1881. Granted leave of absence for twenty-four days from September 24, 1881.

*Purviance, George*, Surgeon. Detailed as member Board of Examiners. September 12, 1881.

*Sawtelle, H. W.*, Surgeon. Detailed as recorder Board of Examiners. September 12, 1881.

*Godfrey, John*, Passed Assistant Surgeon. To proceed to Pascagoula, Miss., as inspector. July 27, 1881.

*Goldsborough, C. B.*, Passed Assistant Surgeon. To proceed to Havre de Grace, Md., as inspector. July 27, 1881. Granted leave of absence for thirty days from September 1, 1881.

*Cooke, M. P.*, Assistant Surgeon. Granted leave of absence for thirty days from August 12, 1881.

*Carter, H. R.*, Assistant Surgeon. Granted leave of absence for eight days from September 24, 1881.

